# 

**Published:** 1881-04

**Authors:** 


PAGE.

ORIGINAL COMMUNICATIONS:

Cremation. By Frederick Peterson, M. D. 385
The Treatment of Ectropion by Transplantation of Skin. By Dr. Lucien Howe, . 398
The Heatonian Method for the Radical Cure of Hernia. By W. H. Heath, M. D., - 405

CLINICAL REPORTS:

Surgical Cases Reported by C. C. Frederick, House Physician, . . . . . 412–414
Prof. Moore's Clinic at Buffalo General Hospital. Reported by Dr. Peterson, - - 414

TRANSLATIONS:

Some Additional Experiments upon the Communicability of Tuberculosis in Dogs by means of inhalation of Phthisical Sputa. By Dr Tappeiner, at Tueran. From the German by P. W. Van Peyma, M. D. . 418

EDITORIAL:

Vivisection Once More, . . . . . 421
Registered Practitioners, . . . . . 423
In Memoriam—Death of Horatio N. Loomis, M. D., . . . . . . . . 424

REVIEWS, . . . . . . . 425

